# Characterization and Valorization of *Agave* Bagasse for the In Vitro Growth of *Pleurotus agaves*

**DOI:** 10.3390/polym18070834

**Published:** 2026-03-29

**Authors:** Alejandra Valdez-Betanzos, Rosalva Mora-Escobedo, Gerardo Mata-Montes de Oca, Humberto Hernández-Sánchez, José Antonio Guerrero-Analco

**Affiliations:** 1 Departamento de Ingeniería Bioquímica, Escuela Nacional de Ciencias Biológicas, Instituto Politécnico Nacional, Ciudad de México 07738, Mexicohhernan1955@gmail.com (H.H.-S.); 2 Red de Manejo Biotecnológico de Recursos, Instituto Nacional de Ecología, Asociación Civil (A.C), Xalapa 91073, Veracruz, Mexico; 3 Red de Estudios Moleculares Avanzados, Instituto Nacional de Ecología, Asociación Civil (A.C), Xalapa 91073, Veracruz, Mexico; joseantonio.guerrero@inecol.mx

**Keywords:** edible mushrooms, *Agave angustifolia*, *Agave marmorata*, *Agave potatorum*, lignocellulosic residues

## Abstract

Sustainable revalorization of agave bagasse, a lignocellulosic residue from mezcal production, is essential for environmental management. This study evaluated its potential as a substrate for the in vitro cultivation of the wild edible mushroom *Pleurotus agaves*. Characterization revealed a robust lignocellulosic matrix (70.9–75.87% NDF, 42.05–51.18% ADF and 10% lignin) and significant antioxidant potential, particularly in *A. marmorata*, which also exhibited higher total reducing sugars (11.94 mg/mL). This provides an energetic advantage for initial mycelial growth. Substrate microstructure was analyzed via microscopy (CLSM/SEM) before and after thermal pretreatment (55 °C). The IE-2038 strain was tested in five formulations: straw (P-55), bagasse (B-55), and straw–bagasse mixtures at 50–50%, 25–75%, and 75–25%. Mycelial growth rates indicated that PB-55 and pB-55 exhibited the fastest fungal colonization (8.2 mm/day and 8.3 mm/day). Microstructural analysis revealed significant damage to the polymeric organization of the bagasse, caused by mezcal production techniques and thermal treatment. This damage made lignin and cellulose more accessible for *P. agaves*. This synergy is supported by the adaptation of *P. agaves* to agave stalks. These findings confirm the capacity of bagasse as a sustainably bioprocessed substrate for edible mushroom cultivation, providing an effective alternative for the revalorization of agro-industrial residues that contribute to the circular economy.

## 1. Introduction

Recently, the search for and implementation of sustainable management alternatives for agro-industrial residues have scaled globally and have affected various sectors, including the alcoholic beverages industry. In Mexico, one such sector is mezcal, a traditional spirit of significant cultural and economic importance protected by a designation of origin whose production has experienced continuous growth, resulting in a massive generation of residues, such as bagasse.

This by-product is the final residue from the sugar extraction processes of the *Agave* pineapples (heads); it is the set of fibrovascular bundles and husk that comprise the maguey head, which is composed primarily of lignocellulosic fibers. Bagasse represents approximately 40% of the wet weight of milled maguey [1,2,3]. Generation rates are approximately 4 to 12 kg per liter of mezcal, depending on the *Agave* species. According to production data from the Mexican Mezcal Regulatory Council [4], this amount could translate to a generation of 55,390 tons of bagasse in 2024 in the state of Oaxaca. Its abundance complicates handling, transportation, storage, and treatment, resulting in illegal dumpsites that are commonly identified in plots or farmlands, disposal in rivers and streams, or accumulation outside or around the palenques (mezcal distilleries). Furthermore, the lignocellulosic nature of bagasse hinders its degradation by environmental microorganisms [5].

Bagasse is a valuable material because its main composition is a complex matrix of natural polymers, including cellulose (59.3%), hemicellulose (15.4%), and lignin (17.2%), in addition to compounds with biological activity. However, its nature and highly organized structure render bagasse a recalcitrant material, making its degradation by ambient microorganisms difficult. This challenge has created numerous opportunities within the biotechnological sphere [6,7].

The valorization of bagasse has resulted in various applications, such as the production of biofuels [8,9], animal feed and forage [10,11,12], a substrate for agricultural crops [13,14], and even in food production as an additive in baking flours [15]. In addition to these applications, one of the most promising technologies is the cultivation of edible mushrooms from the genus *Pleurotus*. These fungi are characterized by their role as efficient biological degraders because of their ability to secrete enzymes that are capable of breaking the lignocellulosic matrix and transforming the residue into a nutritious food product [16].

Despite the versatility of this genus, research specifically focused on *P. agaves,* a wild edible species traditionally gathered and consumed by communities in Mexico [16], is extremely limited. This species represents a valuable nutritious food product with bioactive compounds. However, its transition from wild gathering to controlled cultivation has not been fully established. Unlike commercial strains, *P. agaves* possesses a natural affinity for the *agave* chemical matrix, as it grows on *Agave salmiana* stalks (pencas); nevertheless, its cultivation on mezcal bagasse remains unexplored.

Notably, the efficiency of fungal degradation largely relies on fungal access to cellulose. Therefore, the application of pretreatments and the adjustment of conditions such as substrate pH are crucial for destroying the polymeric matrix and increasing fungal growth [3]. Nevertheless, a knowledge gap persists regarding the effect of initial mezcal production processes and the efficacy of pretreatments on different *Agave* species (such as *Agave angustifolia*, *Agave potatorum*, and *Agave marmorata*). More critically, the relationship between process optimization and the microstructural damage of a polymer is rarely visualized and quantified using advanced techniques.

Therefore, the objective of this study was to evaluate the potential of *agave* bagasse as a substrate for *P. agaves* through a three-stage approach: first, determining the initial physicochemical characteristics of the bagasse to identify its limiting factor; second, performing an in vitro evaluation to determine the baseline fungal growth; and third, was implementing and validating a thermal pretreatment to overcome substrate recalcitrance and enhance mycelial development.

## 2. Materials and Methods

Air-dried bagasse from three *agave* species was used: *A. angustifolia* (obtained from a mezcal distillery, or palenque, located in Santiago Matatlán, Tlacolula, Oaxaca) and two wild *agave* species, *A. marmorata* and *A. potatorum* (obtained from a palenque located in San Baltazar Guelavila, Oaxaca).

### 2.1. Physicochemical and Proximal Analysis

The moisture content [17] (AOAC 931.04, 1990), ash [18] (AOAC 942.05, 1990), crude protein (micro-Kjeldahl method, AOAC 988.05, 1990) [19], and fat or ethereal extract (Soxhlet method, AOAC 920.85, 1990) [20] were determined. The total carbohydrate content was calculated by the difference. All physicochemical, proximal, and antioxidant capacity determinations were performed in triplicate (*n* = 3).

### 2.2. Lignocellulosic Polymer Quantification (NDF, ADF and ADL)

Lignocellulosic materials were determined according to the Van Soest methodology (1967) [21]. This approach involved quantifying the neutral detergent fiber (NDF), which comprises the cell wall fibers composed of cellulose, hemicellulose, and lignin; acid detergent fiber (ADF), which comprises the cell wall fibers composed of cellulose and lignin; and acid detergent lignin (ADL). The fraction was extracted from the ADF residue using 72% sulfuric acid to dissolve the cellulose.

All determinations were calculated on a dry weight basis and corrected using the blank. Additionally, the differences in the contents of cellulose and hemicellulose were calculated. The following formulas were used to calculate the three primary fractions:

(1) $$\%Fraction\;=\;100\;*\;\frac{\left(weight\;of\;residue-ashes\right)-weight\;of\;blank}{Inicial\;sample\;weight\;(g)}$$

After the NDF, ADF and ADL data were obtained, the quantification of cellulose and hemicellulose was performed as follows:

Cellulose content = ADF − ADL

Hemicellulose content = NDF – ADF

### 2.3. Determination of Polyphenols and Antioxidant Activity in A. angustifolia, A. marmorata, and A. potatorum

#### 2.3.1. Preparation of Extracts

The bagasse samples were pulverized in a blade mill (Hamilton Beach 80350R) (80350R; Hamilton Beach, Glen Allen, VA, USA) and subsequently sieved to a specific particle size of 1 mm. The samples were then subjected to alcoholic maceration: 1 g of the pulverized sample was combined with methanol:water (80:20) and left under agitation at ambient temperature for 24 h. The extract was obtained after double centrifugation (14 pro; Dynamica scientific centrifuge Ltd., Livingston, UK) at 2795× *g*. The resulting supernatant was concentrated to dryness using a rotary evaporator (R-300; BÜCHI Labortechnik AG, Flawil, Switzerland) and then resuspended in 15 mL of distilled water for storage in amber glass vials under refrigeration until use.

#### 2.3.2. Determination of Total Polyphenols in *A. angustifolia*, *A. marmorata*, and *A. potatorum*

The total polyphenol content was quantified according to the Folin–Ciocalteu method (Singleton and Rossi 1965, with modifications by Bobo-García et al., 2015) [22]. The absorbance was read at 750 nm in a microplate reader (Multiskan 60; Thermo Fisher Scientific, Waltham, MA, USA) after a 2 h incubation period with the Folin–Ciocalteu reagent (Sigma-Aldrich, St. Louis, MO, USA) and a 10% sodium bicarbonate solution (Sigma-Aldrich, ST. Louis, MO, USA). The results are expressed as micrograms of gallic acid equivalents per mL of sample (µg GAE/mL) on the basis of a previously constructed gallic acid standard curve (considering concentrations of 0.012, 0.010, 0.008, 0.006, 0.004, 0.002 and 0.000 mg/mL which yielded a linearity coefficient R^2^ of 0.9975).

#### 2.3.3. Determination of the Antioxidant Capacity of *A. angustifolia*, *A. marmorata* and *A. potatorum*

Antioxidant activity was determined using DPPH [22] and ABTS [23] radical-scavenging assays, both of which were performed in a microplate format. The reaction with the methanolic DPPH solution (150 µmol/L) was read at 515 nm using a microplate reader (Thermo Fisher Multiskan 60) after 40 min of incubation in the dark. The results are expressed in µmol Trolox equivalents. Quantification was based on a previously established Trolox calibration curve prepared in methanolic solution (methanol:water ratio of 80:20). The curves for concentrations of 0, 50, 100, 150, 200 and 250 µmol/L yielded a linear coefficient R^2^ of 0.9966. The ABTS radical was prepared by reacting a 7 mmol/L ABTS stock solution with 2.45 mmol/L potassium persulfate. This mixture was incubated for 12–16 h in the dark at ambient temperature. The working ABTS solution was prepared by diluting the stock solution until its absorbance was adjusted to 0.700 at 754 nm. The assay was initiated by adding 50 µL of the sample extract to 250 µL of the diluted ABTS solution. The absorbance at 754 nm was measured at 6 min in a microplate reader (Thermo Fisher Multiskan 60). The results are expressed in mg Trolox equivalents per gram of dry weight (mgTE/gDW). Quantification utilized a previously established Trolox calibration curve with concentrations of 0, 50, 100, 150, 200 and 250 µmol/L and a linearity coefficient R^2^ of 0.9982.

### 2.4. Microstructural Characterization in A. angustifolia, A. marmorata, and A. potatorum

Microstructural analysis of bagasse fibers from *A. angustifolia*, *A. marmorata*, and *A. potatorum* was performed to characterize their structure under two conditions: immediately after the mezcal processing (raw) and after being subjected to thermal pretreatment (55 °C) before fungal inoculation.

#### 2.4.1. Confocal Laser Scanning Microscopy (CLSM)

Confocal laser scanning microscopy (CLSM) is a useful technique for obtaining information about the concentration and spatial distribution of elements within a sample, which are typically stained with fluorochromes [24]. Furthermore, CLSM is non-destructive, and in the case of fibers, it allows 3D images to be obtained from any point of view [25,26].

Bagasse fibers were analyzed using CLSM to characterize the distribution and presence of lignin and cellulose through differential staining. The fibers were fragmented and stained with a solution of calcofluor White M2R (fluorescent brightener, 28, F3543; Sigma Aldrich, St. Louis, MO, USA) for 5 min to label the cellulose. With respect to lignin, natural autofluorescence was observed. The samples were analyzed using a confocal laser scanning microscope (LSM 710, Carl Zeiss, Jean, Germany) with a diode laser (continuous wave/pulsed) at an emission wavelength of 405 nm for cellulose and an argon laser at 488 nm for lignin.

#### 2.4.2. Scanning Electron Microscopy (SEM)

Scanning electron microscopy (SEM) allows images to be obtained with magnifications of up to 500,000 times and a high resolution of less than 1 to 20 nanometers, making it a useful tool for structural characterization [27].

This tool was used to identify and characterize the surface structure and morphological changes of the fibers from *A. angustifolia*, *A. marmorata*, and *A. potatorum,* both untreated and after being subjected to thermal treatment. A variable pressure scanning electron microscope (Quanta 250 Feg; FEI Company, Hillsboro, OR, USA) was used in low-vacuum mode.

### 2.5. Evaluation of Mycelial Growth in an Organic Substrate In Vitro of Pleurotus agaves (Maguey Mushroom)

The evaluation of mycelial growth was divided into two phases: the selection of the *P. agaves* strain with the best adaptation to the substrates and the establishment of the substrate conditions through thermal pretreatment.

#### 2.5.1. First Phase: Selection of the Best-Adapted Strain

Six strains of *P. agaves*, which were obtained from the culture collection of the Unit of Biotechnology of Edible and Medicinal Mushrooms at the Instituto de Ecología, A.C., and identified as IE-715, IE-835, IE-836, IE-837, IE-862, and IE-2038, were evaluated. These strains were maintained on solid potato dextrose agar (PDA) culture medium (Bixon, West Chester, OH, USA) at 25 °C. The inoculum was prepared using sorghum grains (*Sorhum vulgare* L.) according to the methodology of [28].

Wheat straw (positive control) and bagasse of *A. angustifolia* were used as substrates because of their greater availability and accessibility. They were fragmented to a length of 1.5 cm. The substrates were hydrated with potable water for 24 h at ambient temperature. Excess water was subsequently removed until the moisture content was between 60 and 70%. The pH of each substrate was determined, and only the pH of the *agave* bagasse was adjusted to 5.5 using 20 g/Kg wet weight Ca(OH)_2_.

In vitro cultivation was performed according to the methodology of [29]. Glass test tubes (22 × 150 mm) were used and filled with substrate up to a 10 cm mark (equivalent to 13 g of wet substrate). The culture conditions utilized three substrate formulations: (a) 100% wheat straw (P), (b) 100% *agave* bagasse (B), and (c) a 50–50% straw–bagasse mixture (PB). The wheat straw (P) served as the positive control to evaluate the adaptation and growth performance of the six *P. agaves* strains. The tubes were sterilized in an autoclave for 90 min at 121 °C (15 psi). They were then allowed to cool for 24 h. Inside a laminar flow hood, 1 g of inoculum from each experimental strain was used for inoculation. The tubes were sealed with a sterile cotton plug to facilitate gas exchange and incubated in the dark at 25 °C. This day was considered Day 0 of incubation. After 48 h of incubation, the tubes were labeled with two longitudinal lines, “A” and “B,” along which the mycelial growth was recorded every 48 h until the substrate was completely covered by the mycelium. For each treatment and strain, five independent replicates were evaluated.

#### 2.5.2. Second Phase: Substrate Optimization with Thermal Pretreatment

This phase was conducted using the selected strain (IE-2038). In vitro cultivation was performed according to the methodology of [29] with modifications to the substrate preparation. The substrates used were fragmented wheat straw and *A. angustifolia* bagasse subjected to thermal treatment. A total of 600 g of wet substrate was divided into 70 g portions into glass jars with plastic hermetic lids. These were maintained in a drying oven at 55 °C, which was registered as Day 0 of the thermal treatment. On days 2 and 4, the substrate was mixed to favor “aerobic fermentation”. After completion of the thermal treatment (day 6), the pH of the bagasse was adjusted with Ca(OH)_2_. The pH values were measured before and at the end of the thermal treatment, as well as after chemical adjustment. The inoculation conditions were the same as those used in phase 1, and for each treatment, five replicates were evaluated. Evaluation treatments were based on the thermally pretreated substrate (indicated by the suffix -55): 100% straw (P-55), which served as the positive control, 100% bagasse (B-55), 50–50% straw–bagasse mixture (PB-55), 25–75% straw–bagasse mixture (pB-55) and 75–25% straw–bagasse mixture (Pb-55).

### 2.6. Statistical Analysis

The experimental design used was a completely randomized design. The data obtained from the physicochemical characterization and the in vitro mycelial growth evaluation were analyzed using a one-way analysis of variance (ANOVA). Prior to ANOVA, the normality of the data and homogeneity of variances were verified. Significant differences between the *agave* species and the applied treatments were determined with a significance level of α = 0.05. In cases where significant differences were observed, Tukey’s honestly significant difference test was applied for multiple comparisons to identify which specific groups differed.

Pearson’s correlation analysis was performed to explore the linear relationship between the total polyphenol content and the antioxidant activity measured by ABTS and DPPH.

Correlation analysis was performed using the Microsoft Excel spreadsheet program. The remaining statistical analyses (ANOVA and Tukey) were performed using Statistica 7 software, version 7 (Dell Inc., Rock, TX, USA).

## 3. Results and Discussion

### 3.1. Chemical Characterization of A. angustifolia, A. marmorata, and A. potatorum

The results of the chemical characterization of the bagasse samples are presented in Table 1. The moisture content significantly differed (*p* ≤ 0.05), with the maximum values observed for *A. marmorata* (3.73%) and the minimum values observed for *A. potatorum* (2.53%). This trend was similar to that previously reported for the same *agave* species [5]. However, these values differ from those reported in other studies, which report moisture contents ranging from 4% to 13% [8,30]. This low content is consistent with the storage and drying conditions of the bagasse used, because the fibers were uniformly spread out at the palenques (distilleries) and left to air dry for one week to prevent fermentation and/or contamination. After the fibers were dry, they were transferred in sacks to the laboratory for analysis.

The percentage of ash was highest in *A. angustifolia* (8.65%), followed by *A. marmorata* (7.06%) and *A. potatorum* (6.35%). Although these values were not significantly different, they fell within the 1% to 15% range reported for other *agave* species [10,31]. These differences can be associated with the efficiency of grinding at the palenque, because mechanical destruction affects the amount of fiber and pith in the bagasse; thus, a higher percentage of pith results in a higher ash content.

The ash content represents the inorganic reservoir essential for fungal physiology; although a specific elemental analysis of the ash was not performed in this study, the growth of *P. agaves* can be explained by the mineral composition reported for *agave* bagasse in the literature. According to the nutritional standards of [32], *Pleurotus* species require a balanced supply of macro- and microelements to sustain mycelial development. In this context Delgadillo et al. [10] reported that *agave* bagasse contains minerals such as phosphorus and potassium in concentrations around 10^−3^ M, acting as cofactors in various enzymatic systems. Furthermore, the presence of calcium in the form of calcium oxalates (a distinctive characteristic of *agave* fibers) plays a crucial role in the fructification process. Regarding trace elements, the literature suggests that the presence of copper and iron in *agave* substrates favors the stability of the laccase enzyme and the synthesis of catalase, respectively [33]. Therefore, based on these reports, the *agave* bagasse used in this study likely provided a balanced mineral profile that sustained the physiological requirements of *P. agaves*.

The type of milling is also related to the degree of trituration. A higher ash content has been reported when a tahona (stone mill, 14.8%) is used than when a mechanical mill (10.69%) is used in terms of the same *agave* species [5]. Therefore, *A. angustifolia* had a lower grinding efficiency than *A. marmorata* and *A. potatorum* did. This finding can be confirmed by evaluating the pith content after milling to rule out that this difference is solely attributed to the natural composition of the wild *agave* species. The ash content is relevant for mushroom cultivation because it reflects the mineral availability for the development of fungi such as *Pleurotus* [31].

The crude protein content did not significantly differ and was low compared with that reported for other *agave* species, such as *tequilana*, *salmiana*, and *weberi*, whose contents ranged from 2.25% to 3.7% [30,31,34]. In terms of *A. angustifolia*, a similar trend was reported by other authors, with a percentage of 1.75% [33]. With respect to *A. marmorata* and *A. potatorum*, there is no available reference literature; therefore, this study is one of the first on the protein content in these wild *agave* species, for which information is scarce. Our findings are consistent with the lignocellulosic nature of *agave* residues, which typically contain less than 5% (usually between 2% and 3%) protein [35]. While a low percentage is not a direct limiting factor for fungal growth, the protein quality of fruiting bodies is affected by the available nutrients in the substrate [36].

*Agave potatorum* had the highest content of fats (3.29%), followed by *A. angustifolia* (2.40%) and *A. marmorata* (1.45%), with no significant differences among them. Despite this finding, these values fall within the range reported for *A. tequilana* (1%) [37] but are higher than those reported for *A. salmiana* (0.274% to 0.84%) and *A. weberi* (0.272% to 0.5%) [10,31]. The quantity of fats is generally low, and their presence is associated with the environmental characteristics of the *agaves* species.

Exposure to stress conditions such as high temperatures and sustained droughts facilitates the synthesis of lipids [33], including terpenes and fatty acids [35]. The fat content is related to the origin of the *agaves* and not the mezcal production process. Therefore, the higher fat content in *A. potatorum* could be associated with its origin; because it is designated a “wild *agave*”, it does not undergo mechanized cultivation; that is, its collection occurs in the field under adverse and natural conditions, which facilitates the synthesis of lipids as a defense mechanism [33]. These levels do not represent an inhibitory factor; on the contrary, they function as a high-density supplementary energy source. While the fungus degrades the lignocellulosic matrix to obtain structural carbon, it can simultaneously metabolize lipids through various metabolic pathways [36], thereby obtaining the necessary energy to sustain the secretion of extracellular enzymes during the early stages of colonization [8].

Although some reports suggest that fat contents near 1% favor a slow decomposition of the substrate [32,33,34,35,36,37,38], the higher levels detected in this study do not represent a limiting factor. Instead, they may serve as a supplementary energy source that *P. agaves* can metabolize.

The total reducing sugar (TRS) content significantly differed (*p* ≤ 0.05). *Agave marmorata* had the highest concentration (11.9 mg/L), whereas *A. angustifolia* (3.99 mg/L) and *A. potatorum* (4.82 mg/L) had lower concentrations. The low TRS values in *A. angustifolia* and *A. potatorum* suggest efficient sugar extraction during mezcal production. In contrast, the high concentration in *A. marmorata* could indicate poor control of temperature or milling, because the extraction of sugars is inversely proportional to the amount of residual sugars in the bagasse [37]. During the cooking of the piñas (*agave* heads), temperature is vital because it induces the breakdown of inulin so that it can be used by the yeasts. Therefore, a low temperature prevents polysaccharide degradation, and a high temperature causes caramelization or the maillard reaction of sugars, which positively affects production yield [39].

While low TRS values are a quality parameter for mezcal production, in the application of bagasse as a substrate for edible mushroom cultivation, the use of substrates only or in proportions adapted to the needs of the fungal strains has been suggested [33]. However, the TRS levels detected in the substrates are key for selectivity; while a high concentration of simple sugars provides initial energy for fungal growth [40], an excess has been associated with the proliferation of microbial competitors to fungi like *Trichoderma* spp. [39,41]. In contrast, species of the *Pleurotus* genus possess a specialized enzyme capable of degrading complex compounds [40]. Therefore, they should be subjected to procedures that reduce the TRS levels, facilitating mycelial development [41,42].

The importance of the contents of reducing sugars and other carbon sources (polysaccharides, monosaccharides, organic acids, amino acids, some alcohols, lignin, and cellulose) in mushroom cultivation lies in their role in fulfilling the energetic and structural functions of fungal cells [43]. This is critical, especially during the first days of cultivation, when the simplest polysaccharides, such as reducing sugars and starch, are used while they are available in the substrate [40]. Compared with the other substrates, the bagasse samples in this study present a low TRS content. With respect to the cultivation of *Pleurotus djamour* on pangola grass with a high concentration of reducing sugars (21.71 g/100 g DW), a higher biological efficiency was reported than that of coffee pulp, which had the lowest TRS content (13.76 g/100 g DW) and the lowest biological efficiency [44]. However, studies that involve bagasse from *A. salmiana* and *A. angustifolia*, used alone and combined with pine, cedar sawdust, and nopal, have shown that biological efficiency increases when the substrate presents a lower content of reducing sugars and lipids [33]. Therefore, the bagasse analyzed in this study provides an adequate quantity for mushroom cultivation, with *A. marmorata* potentially providing the most initial energy to the strains, resulting in greater mycelial growth. This provides *P. agaves* with a competitive advantage in the analyzed bagasse, allowing for a more selective mycelial development by limiting the growth of fungi that require easily assimilable sugars.

### 3.2. Determination of the Fiber Fractions

In nature, white rot fungi such as *P. agaves* rely on enzymatic complexes to degrade lignin, cellulose, and hemicellulose [33,45]. In this study, the fiber fractions evaluated were NDF, which corresponds to the cell wall fibers and presents a rigid structure formed by cellulose, hemicellulose, and lignin [46]; ADF, which is composed of the cellulose and lignin of the cell walls, as well as different amounts of xylans and other components; and lignin acid detergent, which is the indigestible component of fiber [47]. The results are listed in Table 1.

Statistically significant differences (*p* ≤ 0.05) in the contents of NDF and ADF were observed among the bagasse species. The highest contents were recorded in *A. marmorata* (51.18% ADF and 75.87% NDF), followed by *A. potatorum* (44.7% ADF and 70.9% NDF) and *A. angustifolia* (42.05% ADF and 72.13% NDF).

These results fall within the typical range reported for other lignocellulosic substrates used in *Pleurotus* cultivation, such as corn stover (41.78%), unspecified maguey bagasse (42.26%), and pine sawdust (43.06%) [11]. However, these values are slightly lower than those reported for *agave* species such as *A. tequilana*, whose NDF contents range from 55% to 58%, whose ADF contents range from 42% to 47%, and whose ADL contents range from 7% to 32% [48,49], and those of *A. salmiana* and *A. weberi*, whose NDF contents are 45% and 52%, respectively [10].

The content of lignin acid detergent did not significantly differ among the species. Nevertheless, a slightly greater trend was observed for *A. angustifolia* (10.83%) and *A. marmorata* (10.43%). The high concentrations of NDF and ADF confirm that the bagasse samples are high-potential substrates for the cultivation of *P. agaves*. It is important to note that while *agave* bagasse is a major source for cellulose extraction due to its high polymer content, its biological conversion through fungal cultivation offers a sustainable alternative. Unlike chemical extraction, this process avoids the use of harsh chemicals and transforms the residue into a nutritional and functional food.

Lignin is among the most critical factors, because it acts as a barrier that encapsulates cellulose and hemicellulose [45]. Although the variations in lignin content among the three species were not significant, its constant presence justifies the need for the thermal pretreatment implemented in phase II to increase surface area. The secretion of oxidative enzymes by the *Pleurotus* genus specifically targets this recalcitrant lignin barrier [40], making the cellulose and hemicellulose fractions more accessible for subsequent degradation [49].

Various studies have shown that the productivity of mushrooms is not linearly related to the initial fiber content. However, the specific enzymatic capacity of the strain and the incubation time required to degrade the polymers are important [49,50]. Therefore, it is not possible to generalize the way in which lignocellulosic fungi use each fraction, because this relies on their own enzymatic activity, which is regulated by factors such as oxygen availability, simple carbohydrate levels, and the interaction of the mycelium with the substrate [44,51]. Previous studies have observed that *Pleurotus* inoculation improves the degradation of fibrous fractions (NDF and ADF) in agricultural residues such as corn (77.4% NDF and 62.2% ADF) and sugarcane (70.4% NDF and 49.9% ADF) [52]. In this sense, the bagasse samples evaluated in this study follow this trend (70.9–75.87% NDF and 42.05–51.18% ADF). These findings suggest that the optimal composition for *P. agaves* is not a static value but resides in its capacity to degrade matrices with high fiber availability.

Because *A. mamorata* has the highest NDF and ADF contents, it is an energetically superior substrate once *P. agaves* overcomes the initial lignification barrier. Therefore, the evaluation of these fiber fractions allows an understanding of the mechanism by which the fungus must act to use the substrate components and how this affects its growth.

### 3.3. Evaluation of Antioxidant Activity

The content of phenolic compounds and the capacity to neutralize free radicals attract great interest owing to the retention of these compounds in lignocellulosic residues. The results are presented in Table 2.

No statistically significant differences in total polyphenol content were detected among the bagasse samples (*p* > 0.05). However, a greater trend was observed for *A. marmorata* (4.06 mg/GAE/g) and *A. potatorum* (2.75 mg/GAE/g) than for *A. angustifolia* (1.23 mg/GAE/g). These results are consistent with those of previous reports for *A. angustifolia* bagasse fibers, residue from a fructan extraction process via leaching (0.91 mg/GAE/g) and *A. angustifolia* bagasse from a mezcal factory (2.65 mg/GAE/g) [53]. However, these values are lower than those in other reports, such as those of Ocampo-Batista et al. (2024) [54], who reported values of 2782.12 to 11,159.12 µgEAG/g for free phenols and 12,094.14 to 20,582.44 µgEAG/g for bound phenols.

On the basis of our results, the polyphenol concentration is similar to that of other important food sources, such as fresh blueberries (358.7 mgEAG/100 g) [55], although it is lower than that of blackberries and raspberries (9.67 to 17.81 mgEAG/g) [56] or native mexican blue corn flour (10.68 to 17.14 mgEAG/g) [57]. Despite this finding, bagasse extracts could be promising sources of bioactive compounds [58].

These secondary metabolites are linked to a response to drought stress [59], and in terms of plant leaves, their content is affected by location and sun exposure [60]. In terms of bagasse, the polyphenol content is associated primarily with its lignocellulosic composition, which is characterized by a high lignin content. Lignin itself is a phenolic polymer that is composed of three main monolignols: p-coumaryl alcohol, sinapyl alcohol, and coniferyl alcohol [53,61]. Species of *Pleurotus* are efficient at degrading these structures through the secretion of laccases, which oxidize phenolic compounds, potentially using them as enzymatic inducers to facilitate the colonization [40].

Antioxidant capacity was evaluated using two methods based on the ability to stabilize synthetic radicals (ABTS and DPPH). With the DPPH assay, statistically significant differences were observed (*p* < 0.05), and *A. marmorata* had the highest activity (1467.68 mgET/g), followed by *A. potatorum* and *A. angustifolia* (855.50 and 769.25 mgET/g, respectively). In terms of the ABTS assay, the results ranged within a narrow band (377.73 to 393.30 mgET/g), with no significant differences among the species (*p* > 0.05). The variability observed in the results compared with previous reports may be related to the nature of the analyte, the solvent, and the extraction conditions [61,62,63]. Nevertheless, antioxidant activity is directly related to the content of phenolic compounds, because their structure is characterized by the presence of a hydroxyl group and double bonds, which allows for the neutralization of free radicals by donating electrons or hydrogens to radical species [53,60].

The Pearson correlation coefficient was evaluated, revealing a positive correlation (*p* = 0.8) between the total polyphenol content and the antioxidant activity measured by the DPPH assay. This finding indicates that total polyphenols are the primary compounds responsible for the capacity to stabilize the DPPH radical. The capacity for this antioxidant activity in bagasse may be affected not only by the amount of lignin in the fibers but also by the mezcal distillation process itself, which could alter or extract specific phenolic compounds contained within the matrix [53]. In the case of the antioxidant activity measured by ABTS, the correlation was negative (*p* = −0.2), which could indicate the interaction of other compounds in the mechanism. This ability of *Pleurotus* to thrive despite the presence of reactive phenolic units underscores its robust enzymatic system during the bioconversion of *agave* residues.

### 3.4. Microstructural Characterization

Microstructural analysis using confocal laser scanning microscopy (CLSM) and scanning electron microscopy (SEM) allowed visualization of the effect of mezcal processing thermal pretreatment (55 °C) on the lignocellulosic matrix, justifying its accessibility for *P. agaves*.

**Figure 1:**
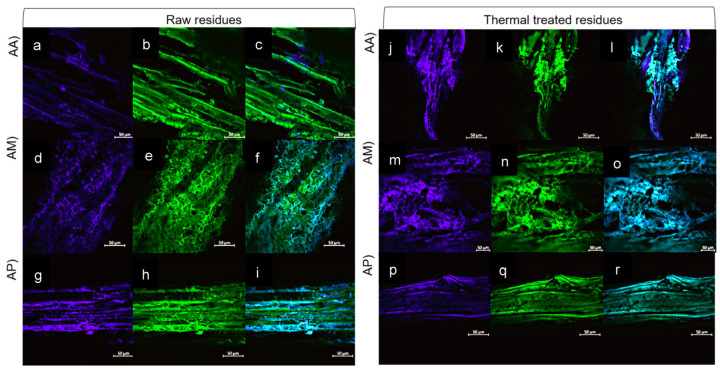
Confocal laser scanning microscopy (CLMS) images of bagasse fibers from Agave angustifolia (AA), Agave marmorata (AM) and Agave potatotrum (AP), showing the distribution of cellulose (**a**,**d**,**g**,**j**,**m**,**p**), lignin (**b**,**e**,**h**,**k**,**n**,**q**) and cellulose–lignin (**c**,**f**,**i**,**l**,**o**,**r**) in the raw state and after heat treatment at 55 °C.

CLMS differentiated between cellulose (stained blue) and the autofluorescence of lignin (green). In terms of the raw bagasse (immediately after mezcal processing), a lower fluorescence intensity was observed for cellulose than for lignin, which was predominant in all three *agave* species. This arrangement revealed a type of lignin encapsulation of the cellulose, especially in raw *A. angustifolia K* (AA) (Figure 1). This coating includes hemicellulose and allows the fibers to remain rigidly joined, resulting in the formation of fibers [64,65] that, although partially modified by industrial cooking and milling processes, still maintain a recalcitrant structure. Furthermore, the observed depolymerization could potentially enhance the antioxidant properties of the bagasse by liberating phenolic functional groups [64], a characteristic that warrants further investigation.

After thermal pretreatment, the fibers exhibited a slight increase in cellulose intensity and a decrease in lignin intensity. In terms of the lignin–cellulose interaction in *A. angustifolia* (AA), an increase in cellulose staining is noticeable. These changes could be associated with an increase in cellulose availability resulting from the action of fungi and microorganisms during thermal treatment (Figure 1). It establishes the basis for *P. agaves* to overcome the recalcitrance barrier by finding more exposed cellulose.

The fibers contain more lignin than cellulose, and in the cultivation of edible mushrooms, this initial process of unwrapping is crucial. With respect to the growth of *Pleurotus*, the degradation of simple compounds such as hemicellulose precedes the degradation of lignin to obtain energy. After these energy sources are depleted, the fungus uses enzymes such as laccase to oxidize the phenolic compounds in lignin, resulting in the production of fruiting bodies [66,67,68,69]. This structural preparation establishes the basis for future production stages by allowing the fungus to overcome the recalcitrant barrier.

**Figure 2:**
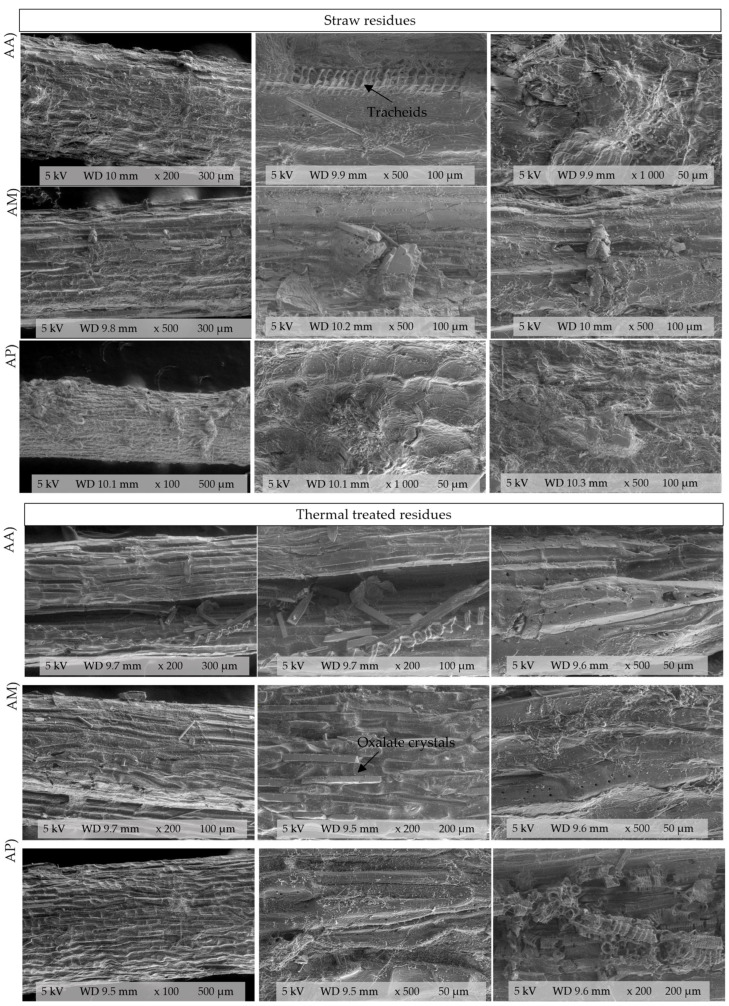
SEM images of bagasse fibers from *Agave angustifolia* (AA), *Agave marmorata* (AM) and *Agave potatorum* (AP), in a raw state and after heat treatment at 55 °C.

The SEM images reveal that the fiber structure is longitudinal (Figure 2). The raw fibers were grouped into fibrous bundles with a rough, compact surface and were characterized by a lack of visible porosity. Signs of damage, such as tears and fractures, were also evident, possibly from the mechanical milling process and the high cooking temperatures during mezcal production [70].

After treatment, surface roughness, porosity and microfissures of the treated fibers increased. This morphological disruption indicates a softening of the fiber, possibly because of the molecular rupture of hemicelluloses and celluloses induced by the temperature [71] and native microbiota, that provides physical entry points for the mycelium and increases the surface area for enzymatic attack. Modifying the physical structure and increasing porosity are critical steps in reducing recalcitrance in complex agro-industrial residues.

Under both conditions (raw and treated), elongated and pointed structures, identified as calcium oxalate crystals known as styloids, were observed. This presence is related to the uptake of soil salts and has been widely reported in these plants [72]. Greater visibility and exposure after thermal treatment were observed in *A. marmorata*, and this could be a secondary effect of the rupture of the fibrous matrix, which highlights the chemical and physical complexity of *agave* residues.

The presence of these hydrophobic compounds is characteristic of *agave* fibers. The microstructural disruption observed after the thermal pretreatment (55 °C) suggests a modification of the cuticle’s lipid layer, which may increase surface accessibility. This is further evidence by the presence of native mycelia and microorganisms observed in the treated fibers. While these correspond to unidentified native microbiota, their presence confirms that the thermal treatment successfully prepared the lignocellulosic matrix, facilitating biological access and the degradation of the parenchyma.

In terms of all three bagasse samples, under both CLMS and SEM, helical coiling of microfibrils is observed. These microfibrils are bundled to create spiral tracheids, which are also visible in the treated fibers. Tracheids are sap-conducting and water-carrying structures composed of lignin and hydrophobic compounds [73]. Thus, visibility could be associated with the destruction of the parenchyma resulting from the high temperatures to which the piñas (*agave* heads) are subjected [53] and their increased exposure to the pretreatment temperature. Furthermore, signs of damage are visible in all three bagasse samples, including tears, scratches, fractures, and a disruption of the septal cells, resulting from the mezcal production process [70]. The surface and structural morphological differences among *agave* species are affected by the processes they undergo [73]. In this study, the residues were collected from various mezcal factories; therefore, a degree of damage and structural differences are evident across the three bagasse types, because each mezcal producer adheres to a specific process. Additionally, as observed, each fiber bundle has unique morphological characteristics, which can affect its physical properties. These differences are attributed to factors such as maturity, the component of the *agave* analyzed, the *agave* variety, the extraction process, and the growth conditions (climate, soil, type, harvest age and species) of the plant [70].

Owing to the complexity of its structure, bagasse is resistant to attack by microorganisms such as fungi; therefore, this factor must be considered. Subjecting the substrates to a prior thermal treatment can facilitate their utilization and accelerate their degradation by allowing them to access cellulose and hemicellulose as a consequence of partial degradation by other microorganisms and fungi [71].

The CLMS and SEM images show the presence of these microorganisms after thermal pretreatment, as in the case of *A. angustifolia*, where microorganisms and the presence of mycelia corresponding to fungi are visible. However, these microorganisms were not identified in this study.

### 3.5. In Vitro Mycelial Growth on the Organic Substrate

The evaluation of in vitro mycelial growth was divided into two phases.

#### 3.5.1. First Phase

This phase focused on the selection of the *P. agaves* strain with the best adaptability, utilizing only *A. angustifolia* bagasse because of its higher presence and viability in terms of quantity. The results of the in vitro growth rate are presented in Table 3.

The IE-2038 strain showed the greatest mycelial growth within 11 days across all three substrates, followed by IE-715 and, subsequently, IE-862. These findings establish IE-2038 as the strain with the best adaptability and vigor to degrade the lignocellulosic substrates of *A. angustifolia*, resulting in its selection for the preparation phase (second phase).

With respect to the substrate, the control substrate (wheat straw) showed the greatest growth, followed by the straw–bagasse mixture (PB) and finally the pure *A. angustifolia* bagasse (B). This lower performance of pure bagasse (B) is consistent with the high recalcitrance observed in the physicochemical analyses, the dense structure, the initial acidic pH (prior to adjustment), the total reducing sugar content, and the presence of oxalate crystals, among other factors related primarily to its nature that hinder the access and activity of fungal enzymes.

This conclusion is supported by the mycelial characteristics: a fluffy or cottony mycelium with a tendency to cover the upper walls of the tubes was observed in many of the samples. This finding could indicate that the strains were experiencing stress because of a deficiency of oxygen, nutrients, or a need to increase the C/N ratio in the substrate.

The change in bagasse coloration after being invaded by the fungus, shifting from a dark color to a brownish hue, confirms the degrative activity of *P. agaves*. This change is largely achieved through the secretion of enzymes that oxidize the lignin and hemicellulose matrix.

#### 3.5.2. Second Phase: Substrate Preparation with Thermal Pretreatment

Owing to the low colonization rate observed in the pure bagasse, the substrates were subjected to a thermal pretreatment at 55 °C for 6 days, followed by a pH adjustment using Ca(OH)_2_.

**Figure 3:**
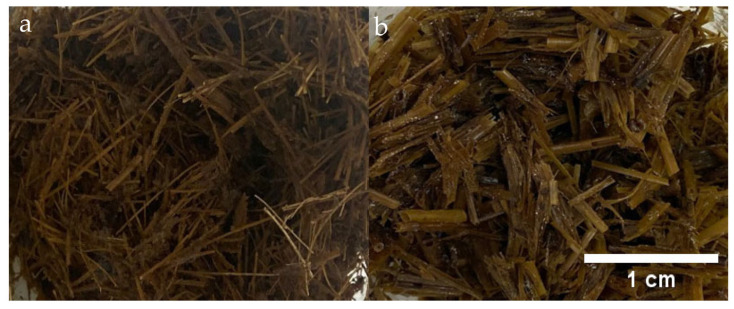
Effect of heat treatment at 55 °C on selective aerobic fermentation and predegradation of lignocellulosic compounds in (**a**) *Agave angustifolia* bagasse and (**b**) wheat straw.

After the thermal treatment, the presence of aerial mycelia was observed on both substrates (Figure 3), which was more notable in the straw than in the bagasse. This finding indicates that a temperature of 55 °C facilitated a type of selective aerobic fermentation of the microbial flora, which was capable of initiating a previous degradation of the lignocellulosic compounds.

The initial pH of the bagasse was strongly acidic (4.59) and increased slightly to 4.86 after the thermal treatment. The addition of Ca(OH)_2_ effectively increased the bagasse pH to 6.87, which is considered optimal for the development of *Pleurotus*. In the case of straw, its alkalinity increased after the thermal treatment, increasing from an initial pH of 7.78 to 9.30 after the thermal treatment.

The results for the growth rate of the IE-2038 strain, presented in Table 4, reveal statistically significant differences between the thermally treated and untreated substrates. The mixtures Pb-55 (75% straw–25% bagasse) and PB-55 (50% straw–50% bagasse) resulted in the highest mycelial growth rates, at 8.3 mm/day and 8.2 mm/day, respectively, indicating that these mixtures are the best substrates. These results are consistent with findings for *Pleurotus djamour* on corn stover and banana leaves, where growth rates have been reported between 11 and 12 mm/day [72], and for *P. eryngii*, which reaches a growth rate of 12.07 mm/day on conventional media and 9.79 mm/day on alternative substrates like corn flour [74].

Although the growth rates achieved in our *agave*–straw mixtures are slightly lower than those reported, they meet the rapidly invasive criterion. This is an essential factor in mushroom biotechnology to ensure biomass production in short periods and prevent the proliferation of competitive contaminants in the substrate [74]. Furthermore, our results are superior to the growth observed on barley straw, which ranges from 3 to 4 mm/day [72].

This vigorous development aligns with observations in specialized media such as potato dextrose agar supplemented with soybean stubble and corn cob, where *Pleurotus* species demonstrated a high capacity to metabolize complex lignocellulosic compounds efficiently [75].

The final mixture, pB-55 (25% straw–75% bagasse), resulted in a growth rate of 7.6 mm/day. These results clearly indicate that thermal pretreatment at 55 °C had a beneficial effect on *agave* bagasse, because *P. agaves* growth on this residue increased from 6 mm/day in B (100% raw bagasse) to 6.8 mm/day in B-55 (100% pretreated bagasse). This increase in growth rate is directly linked to structural disruption evidenced in Section 3.4; the partial degradation of the lignin–cellulose matrix and the pH adjustment allowed *P. agaves* to access the fermentable sugars. The ability of *P. agaves* to develop on this substrate is consistent with observations by [76], who highlighted that the efficiency of *Pleurotus* spp. depends on the prior exposure of fermentable polymers through treatments that partially degrade the lignin barrier.

Conversely, the opposite occurred with straw, where the fungus colonized the untreated substrate P (7.2 mm/day) faster than it colonized the untreated substrate P-55 (6.8 mm/day). This trend has been observed in mushroom production, where a shorter production cycle was observed for unfermented barley straw (58 days) than for fermented barley straw (66 days) [77].

The effect of the thermal treatment on the substrates proved beneficial for the bagasse. This benefit could be attributed to the combination of structural disruption confirmed by the images obtained via SEM and CLSM, as well as pH control, which was crucial for overcoming its recalcitrance. These findings highlight that implementing thermal pretreatment can be a measure to increase the viability of highly lignified and acidic residues such as mezcal *agave* bagasse, facilitating the enzymatic activity of the fungus.

Conversely, the effect on the straw was negative. This finding may be related to changes in its structure and composition because of the action of the bacterial microbiota and is primarily linked to the C:N ratio, which tends to decrease during treatments such as fermentation, and the reduction in sugars caused by high temperatures.

## 4. Conclusions

The results validate the potential of bagasse, the lignocellulosic residue from mezcal production, particularly from the wild species *A. marmorata* and *A. potatorum* (which are poorly investigated), and *A. angustifolia* as a substrate for the cultivation of edible white rot fungi of the genus *Pleurotus*.

The chemical characterization of the three bagasse types confirmed that they contain the essential nutrients required for the growth and development of *P. agaves*. Furthermore, the quantification of phenolic compounds and antioxidant activity suggest the potential for double valorization of these residues.

Despite the previously mentioned findings, and owing to its structurally recalcitrant nature (high lignification and acidic pH), increasing the pH of bagasse and subjecting it to prior thermal treatment at 55 °C was necessary and beneficial. This goal is to induce initial microbial and fungal growth, which degrades complex structures and allows for greater nutrient availability, resulting in the disorganization of the lignocellulosic matrix observed with SEM and CLMS. In vitro validation with the IE-2038 strain revealed that mixtures of straw with pretreated bagasse at proportions of 50–50% (PB-55) and 75–25% (Pb-55) increased growth, with the highest mycelial growth rates occurring at 8.2 mm/day and 8.3 mm/day, respectively. This synergy is supported by the fact that *P. agaves* grows naturally on *Agave salmiana* stalks, suggesting an evolutionary adaptation to the complex chemical matrix of *agave* that enhances its degradation efficiency when compared to conventional substrates.

A microstructural study revealed that the differences in the degree of damage and surface characteristics of the fibers are related to the techniques employed in each factory, confirming that the mezcal production process affects the final quality of the bagasse as a substrate.

In terms of future prospects, production-scale tests should be conducted to identify the biological efficiency and productivity of *P. agaves* in PB-55 and Pb-55 mixtures. This approach will establish the optimal cultivation conditions for the reuse of this valuable agro-industrial residue, providing a viable solution to a significant environmental challenge in mezcal-producing regions. This research highlights the potential of mezcal bagasse as a strategic resource within a circular bioeconomy framework. The biotechnological valorization of these residues transforms an environmental liability into a high-value substrate, promoting sustainable production models that reintegrate industrial by-products into the value chain.

## Figures and Tables

**Table 1:** Proximate chemical analysis of bagasse from three mezcal *agave* varieties.

Content (% Dry Matter)			
	*Agave angustifolia*	*Agave potatorum*	*Agave marmorata*
Moisture	2.53 ± 0.57 ^ab^	2.30 ± 0.53 ^b^	3.73 ± 0.38 ^a^
Ash	8.65 ± 1.37	6.35 ± 0.89	7.06 ± 4.01
Protein	2.00 ± 0.22	1.80 ± 0.03	1.35 ± 1.32
Fat	2.40 ± 0.28	3.29 ± 0.86	1.45 ± 0.07
Carbohydrates	86.49	87.00	89.98
TRS (mg/mL)	3.99 ± 0.21 ^c^	4.83 ± 0.55 ^b^	11.94 ± 0.11 ^a^
NDF	72.13 ± 1.48 ^b^	70.9 ± N/A ^b^	75.87 ± 0.42 ^a^
ADF	42.05 ± 0.95 ^c^	44.7 ± 0.74 ^b^	51.18 ± 0.14 ^a^
Lignin	10.83 ± 1.13	9.7 ± 0.78	10.43 ± 0.21
Hemicellulose	62.15 ± 0.35	61.3 ± 0.78	65.7 ± 0.21
Cellulose	30.3 ± 0.57	26.2 ± 0.92	25.3 ± 0.57

All results are reported on a dry basis and represent the mean of three determinations ± standard deviation. Values within a row or column with different superscript letters are significantly different (*p* ≤ 0.05) according to Tukey’s test. The highest value is denoted by the letter “a”. Abbreviations: TRS: total reducing sugars; NDF: neutral detergent fiber; ADF: acid detergent fiber.

**Table 2:** Total polyphenol content and antioxidant activity of mezcal *agave* bagasse.

Assay	Substrate		
	*Agave angustifolia*	*Agave potatorum*	*Agave marmorata*
Total phenolic content	1.23 ± 0.91 mgEAG/g sample	2.75 ± 2.70 mgEAG/g sample	4.06 ± 0.63 mgEAG/g sample
DPPH	769.25 ± 0.88 mgET/g sample ^b^	855.50 ± 64.52 mgET/g sample ^b^	1467.68 ± 4.86 mgET/g sample ^a^
ABTS	390.34 ± 4.32 mg ET/g sample	393.30 ± 25.05 mg ET/g sample	377.73 ± 8.45 mg ET/g sample

Values are means ± standard deviation of 3 replicates. Values with different superscript letters are significantly different, where “a” denotes the highest value, according to Tukey’s test (*p* < 0.05). mgEAG represents milligrams of gallic acid equivalent, mgET denotes milligrams of trolox equivalent and µmol ET indicates micromoles of trolox equivalent.

**Table 3:** Mycelial growth (mm/day) of *Pleurotus agaves* strains on various substrates during 11 days of incubation.

Substrate	Strains						
	IE-2038	IE-715	IE-862	IE-836	IE-837	IE-835	
P	7.2 ± 0.22 ^a^	7.0 ± 0.23 ^ab^	6.6 ± 0.55 ^abc^	6.5 ± 0.67 ^abcd^	5.9 ± 0.18 ^cdef^	6.0 ± 0.84 ^bcde^	6.5 ± 0.7 ^a^
B	6.0 ± 0.53 ^bcde^	5.8 ± 0.45 ^cdefg^	5.0 ± 0.21 ^efghi^	4.8 ± 0.43 ^ghi^	4.7 ± 0.46 ^hi^	4.5 ± 0.85 ^i^	5.1 ± 0.8 ^c^
PB	7.0 ± 0.27 ^ab^	6.2 ± 0.16 ^abcd^	5.6 ± 0.19 ^cdefgh^	5.5 ± 0.23 ^defghi^	4.9 ± 0.41 ^fghi^	4.9 ± 0.33 ^fghi^	5.7 ± 0.8 ^b^
	6.8 ± 0.64 ^a^	6.3 ± 0.58 ^a^	5.7 ± 0.76 ^b^	5.6 ± 0.84 ^bc^	5.2 ± 0.64 ^c^	5.1 ± 0.94 ^c^	

Values are represented as the mean ± standard deviation of five replicates. Values with different superscript letters differ significantly, where “a” represents the highest value, according to Tukey’s test (*p* ≤ 0.05). Substrates are abbreviated as follows: P (100% straw), B (100% bagasse), and PB (50–50% straw–bagasse).

**Table 4:** Effects of thermal pretreatment on the average mycelial growth rate (mm/day) of *Pleurotus agaves* strain IE-2038, evaluated over a 13-day incubation period.

Substrates	Growth Rate (GR)
B-55	6.8 ± 0.08 ^cde^
P-55	6.8 ± 0.11 ^cd^
PB-55	8.2 ± 0.20 ^a^
Pb-55	8.3 ± 0.43 ^a^
pB-55	7.6 ± 0.30 ^abc^
P	7.2 ± 0.22 ^bc^
B	6 ± 0.52 ^e^
PB	7 ± 0.27 ^c^

Values are presented as the mean ± standard deviation of five replicates. Significant differences (*p* < 0.05) between the values are indicated by different superscript letters, where the letter ‘a’ denotes the highest mean, according to Tukey’s test. Substrates without treatment: P (100% straw), B (100% bagasse) and PB (50–50 straw–bagasse). Substrates with treatment at 55 °C: P-55 (100% straw), B-55 (100% bagasse), PB (50–50 straw–bagasse), Pb (75–25 straw–bagasse), pB (25–75 straw–bagasse).

## Data Availability

The original contributions presented in this study are included in the article. Further inquiries can be directed to the corresponding authors.
